# Serum Vitamin B12 and Holotranscobalamin Levels in Subclinical Hypothyroid Patients in Relation to Thyroid-Stimulating Hormone (TSH) Levels and the Positivity of Anti-thyroid Peroxidase Antibodies: A Case-Control Study

**DOI:** 10.7759/cureus.61513

**Published:** 2024-06-01

**Authors:** Muqdad Al-Mousawi, Sherwan Salih, Ameer Ahmed, Barhav Abdullah

**Affiliations:** 1 Laboratory Medicine, Shaikhan General Hospital, Duhok, IRQ; 2 Medical Chemistry, College of Medicine, University of Duhok, Duhok, IRQ; 3 Hematology, College of Medicine, University of Duhok, Duhok, IRQ; 4 Medical Laboratory Sciences, College of Health Sciences, University of Duhok, Duhok, IRQ

**Keywords:** thyroid-stimulating hormone, thyroxine, holotranscobalamin, vitamin b12, subclinical hypothyroidism

## Abstract

Background

Subclinical hypothyroidism (SCH) is characterized by elevated thyroid-stimulating hormone (TSH) levels, while thyroid hormones (free thyroxine (T4) and free triiodothyronine (T3)) remain within the reference ranges. Vitamin B12 (cobalamin) deficiency is common in patients with autoimmune disorders, including autoimmune hypothyroidism. The study was aimed at evaluating serum vitamin B12 levels and holotranscobalamin (HoloTC) levels in SCH patients and ascertaining their association with a risky level of TSH and the positivity of anti-thyroid peroxidase (anti-TPO) antibodies.

Methodology

A case-control study was conducted at Azadi Teaching Hospital, Duhok, a city in the Kurdistan region of Iraq, involving 153 participants, including 72 newly diagnosed SCH patients and 81 healthy controls. Serum levels of vitamin B12, HoloTC, TSH, free T4, free T3, and anti-TPO antibodies were measured based on different principles.

Results

The mean age of patients with SCH was 32.87±8.7 years, with predominantly females comprising 75% and 77.8% being less than 40 years of age. Moreover, the mean levels of serum TSH (6.96±2.68 µIU/L), anti-TPO antibodies (53.31±81.32 IU/ml), and HoloTC (41.93±19.42 pmol/l) were significantly higher in patients with SCH compared to healthy control participants (p < 0.05), whereas there was a non-significantly higher level of vitamin B12(320.72±98.42 pg/ml) among SCH patients compared to healthy control participants (p = 0.220). The mean levels of vitamin B12 (345.33±103.22 pg/ml) and HoloTC (40.14±18.16 pmol/l) were insignificantly lower in SCH patients with TSH levels more than 7 µIU/L (p > 0.05), as well as the mean levels of vitamin B12 (308.82±96.12 pg/ml) and HoloTC (41.14±19.29 pmol/l) insignificantly lower in SCH patients with positive anti-TPO antibodies (p > 0.05).

Conclusions

This study highlights the potential association between SCH and altered vitamin B12 status, particularly evident in HoloTC levels. The presence of positive anti-TPO antibodies and the degree of elevation in TSH levels may exacerbate vitamin B12 deficiency in SCH patients.

## Introduction

Vitamin B12 (cobalamin) is a complex molecule containing a cobalt ion at its center [1]. Vitamin B12 is critical for several metabolic processes in the body, including DNA synthesis, red blood cell formation, and maintenance of neurological function, as it is regarded as an integral or essential part of two essential enzymatic reactions in the body, such as the conversion of methylmalonyl-CoA to succinyl-CoA (L-methylmanlonyl-CoA mutase) and the conversion of homocysteine (Hcy) to methionine (methionine synthase) [2]. As vitamin B12 is mainly synthesized by bacteria in the digestive tracts of animals, its major dietary sources are animal products like meat, fish, eggs, and dairy products [3]. Absorption of vitamin B12 occurs in the small intestine, facilitated by intrinsic factor, a glycoprotein produced by gastric parietal cells of the stomach [4]. In the circulation, vitamin B12 binds to holohaptocorrin and holotranscobalamin (HoloTC). Holotranscobalamin is a beta protein secreted mainly from the liver and is regarded as a bioactive fraction as it has receptor-mediated cellular uptake [5]. Holotranscobalamin accounts for 6%-20% of endogenous plasma vitamin B12 has one binding site for vitamin B12, and is responsible for transporting vitamin B12 to receptors on the cell membrane throughout the body [6]. 

Subclinical hypothyroidism (SCH) denotes a condition where serum thyroid hormone levels (thyroxine (T4) and triiodothyronine (T3)) remain within the reference range while serum thyroid-stimulating hormone (TSH) levels are elevated above the normal range [7]. Subclinical hypothyroidism is a common disorder that affects up to 10% of iodine-sufficient populations, exhibiting higher prevalence rates among women and the elderly [8]. Worldwide, as the majority of patients with SCH are asymptomatic, its diagnosis relies primarily on biochemical parameters (thyroid function tests) [9].

The prevalence of vitamin B12 deficiency in the general population is approximately 3%-4%. Its deficiency is commonly associated with the autoimmune process, with pernicious anemia being the most common cause of vitamin B12 deficiency, as well as being more prevalent among individuals with primary autoimmune hypothyroidism, potentially affecting up to 12% of hypothyroid patients [10]. Moreover, vitamin B12 deficiency in hypothyroid patients can stem from various factors, including inadequate intake or altered intestinal absorption due to factors such as sluggish bowel motility, bowel wall edema, and bacterial overgrowth [11]. These non-autoimmune causes of B12 deficiency in hypothyroid patients have not been extensively studied [12]. The diagnosis of vitamin B12 deficiency is challenging, as it is widely recognized that there is no single marker that stands out as the best for diagnosing vitamin B12 deficiency [13]. Generally, serum levels of vitamin B12, HoloTC, methylmalonic acid (MMA), and Hcy are commonly utilized for the assessment of vitamin B12 status [14]. Holotranscobalamin has been described as the earliest marker of vitamin B12 deficiency, presenting a narrower gray zone and heightened sensitivity and specificity compared to traditional serum cobalamin assays alone [13].

As the availability of data about the association between vitamin B12 deficiency and SCH was limited all over the world and particularly in our locality, the present study was aimed at evaluating the serum levels of both vitamin B12 and its transporter protein HoloTC in SCH patients compared with apparently healthy control subjects and to ascertain the association of mean vitamin B12 and HoloTC with the risky levels of TSH and the positivity of the anti-thyroid peroxidase (anti-TPO) antibodies.

## Materials and methods

The present case-control study was conducted at the Endocrinology and Diabetes Unit of Azadi Teaching Hospital in Duhok, a city in the Kurdistan region of Iraq, over one year from January 2023 to December 2023. A total of 153 participants were enrolled in the study, consisting of 72 newly diagnosed SCH patients attending the Endocrine and Diabetic Unit and 81 apparently healthy individuals as a control group. They were selected from relatives and neighbors for comparison. Patients with a TSH level greater than 4.2 µIU/ml with normal free T4 and free T3 were considered to have SCH after excluding other cases that had the same biochemical abnormalities [14, 15].

A well-prepared questionnaire was distributed to all participants, including questions about name, age, gender, medical and surgical history, and current drug administration (Appendix A). The anthropometric parameters were taken, such as waist circumference (WC), height, and weight, with the calculation of body mass index (BMI) by dividing weight in kilograms by the square of height in meters (kg/m^2^). The patients and healthy subjects were matched for age and gender.

The inclusion criteria included patients experiencing SCH for the first time and not currently undergoing thyroid replacement therapy. Exclusion criteria include patients with a medical history of thyroid disorders such as primary overt hypothyroidism, secondary hypothyroidism, hyperthyroidism, thyroidectomy, radioactive iodine treatment, or hypothyroidism resulting from medications like amiodarone [16]. Moreover, patients with alcoholism, vegetarianism, previous gastrointestinal surgery, pancreatic insufficiency, ongoing vitamin B12 supplementation, prolonged use of metformin or proton pump inhibitors, inflammatory bowel disease, and celiac disease were also excluded as they affected the vitamin B12 status. Pregnant women were ineligible for participation in the study [17, 18].

All the participants (patients and control) were informed to reach the laboratory department (Clinical Biochemistry Unit) of Azadi Teaching Hospital in the morning and after overnight fasting for blood collection. Five ml of venous blood samples were taken from all participants and poured into a gel tube for centrifugation and obtaining serum for analysis and measurement of the following parameters: TSH (0.2-4.2 µlU/mL), free T4 (12-22 pmol/L), free T3 (3.1-6.8 pmol/L), anti-TPO antibodies (up to 34 IU/L), vitamin B12 (200-771 pg/ml), and HoloTC (25-125 pmol/l) depending on different principles. Vitamin B12, TSH, free T4, free T3, and anti-TPO antibodies were measured by Cobas 6000 (Roche Diagnostics, Basel, Switzerland) using the electrochemiluminescence (ECL) immunoassay principle, and HoloTC was measured by enzyme-linked immunosorbent assay (ELISA) in a sandwich enzyme immunoassay form depending on the antigen-antibody reaction and enzymatic reaction.

Ethical permission was obtained from the scientific committee of the College of Medicine at the University of Duhok as well as from the Research Committee of the Directorate of Health in Duhok (approval number: 27032024-2-10).

Statistical analysis

The statistical calculations were executed by IBM SPSS Statistics Software for Windows, version 26.0 (IBM Corp., Armonk, NY). The anthropometric characteristics among study groups (patients and control) were shown as a mean (SD) or as a percentage. Independent t-tests and Pearson's chi-square test were utilized to compare the groups in the study. To evaluate the relationship between anti-TPO antibodies and TSH and vitamin B12 and HoloTC, independent t-tests were performed. A p-value of 0.05 or less is considered statistically significant.

## Results

The differences in anthropometric characteristics between patients with SCH and healthy control participants are showcased in Table 1. The mean age of patients with SCH was 32.87±8.7 years, with females predominant (75%), and 77.8% were less than 40 years of age. Moreover, the mean BMI was significantly higher among patients with SCH (29.42±6.95 kg/m^2^, p = 0.009), and the WC was insignificantly higher (91.78±14.74 cms) compared to healthy controls. 

**Table 1:** Anthropometrics characteristics of the study participants ^a^ Pearson Chi-Square and ^b^ independent T-test were performed for statistical analysis.

Subject characteristics	Patients (n=72)	Controls (n=81)	p-value
	Mean± SD, No%	Mean± SD, No%	
Gender			
Male	18 (25%)	20 (24.8%)	1.000^a^
Female	54 (75%)	61 (75.2%)	
Age (years)	32.87±8.7	32.82±8.54	0.966^b^
<40 years	54 (77.8%)	64 (79%)	1.000^a^
≥40 years	16 (22.2%)	17 (21%)	
Waist circumference (cm)	91.78±14.74	88.80±15.24	0.223^b^
Male <102 cm	15 (20.8%)	14 (17.3%)	0.454^a^
Male ≥102 cm	3 (4.2%)	6 (7.4%)	
Female <88 cm	19 (26.4%)	33 (40.7%)	0.060^a^
Female ≥ 88 cm	35 (48.6%)	28 (34.6%)	
BMI (kg/m^2^)	29.42±6.95	26.85±5.07	0.009^b^
Normal	17 (23.6%)	50 (61.7%)	0.057^a^
Overweight and obese	55 (76.4%)	31 (38.3%)	

The mean levels of serum TSH (6.96±2.68 µIU/L, p<0.001) and anti-TPO antibodies (53.31±81.32 IU/ml, p=0.001) were significantly higher in patients with SCH compared to healthy control participants, as well as the HoloTC levels (41.93±19.42 pmol/l, p=0.021) were significantly lower in patients with SCH compared to healthy control participants. However, there were insignificantly lower levels of vitamin B12 among patients with SCH compared to healthy control participants, as shown in Table 2.

**Table 2:** Biochemical parameters of the study participants Independent T-tests were performed for statistical analysis. TSH: thyroid-stimulating hormone; FT4: free thyroxine; FT3: free triiodothyronine; anti-TPO: anti-thyroperoxidase; HoloTC: holotranscobalamin

Parameters	Patients (n=72)	Controls (n=81)	p-value
	Mean± SD	Mean± SD	
TSH (µIU/L)	6.96±2.68	2.42±0.96	<0.001
FT4 (pmol/l)	15.01±1.98	16.65±2.02	<0.001
FT3 (pmol/l)	3.72±0.56	3.59±0.34	0.075
Anti-TPO antibodies (IU/ml)	53.31±81.32	19.99±25.24	0.001
HoloTC (pmol/l)	41.93±19.42	48.01±12.32	0.021
Vitamin B_12_ (pg/ml)	320.72±98.42	340.84±103.09	0.220

Table 3 shows that the mean levels of vitamin B12 and HoloTC were insignificantly lower in SCH patients with positive anti-TPO antibodies (308.82±96.12 pg/ml, p=0.174; 41.14±19.29 pmol/l, p=0.646), respectively.

**Table 3:** Mean vitamin B12 level and HoloTC level in subclinical hypothyroid patients with positive anti-TPO antibodies Independent T-tests were performed for statistical analysis. anti-TPO: anti-thyroid peroxidase antibody; HOLO TC: holotranscobalamin

Parameters	Anti-TPO antibodies in subclinical hypothyroidism		p-value
	Anti-TPO antibodies <34 IU/ml	Anti-TPO antibodies ≥34 IU/ml	
	n=46 (63.9%)	n=26 (36.1%)	
	Mean± SD	Mean± SD	
Vitamin B12 (pg/ml)	341.77±100.81	308.82±96.12	0.174
HoloTC (pmol/l)	43.35±19.97	41.14±19.29	0.646

Table 4 shows that the mean levels of vitamin B12 and HoloTC were insignificantly lower in SCH patients with TSH levels greater than 7 µIU/L compared to those with TSH levels less than 7 µIU/L (345.33±103.22 pg/ml, p=0.135; 40.14±18.16 pmol/l, p=0.272), respectively.

**Table 4:** Mean vitamin B12 level and HoloTC level in subclinical hypothyroid patients with TSH level ≥7.0 µIU/L and less than 7 µIU/L Independent T-tests were performed for statistical analysis. TSH: thyroid-stimulating hormone; HoloTC: holotranscobalamin

Parameters	TSH in subclinical hypothyroidism		p-value
	TSH <7.0 µIU/L	TSH ≥7.0 µIU/L	
	n=48 (66.6%)	n=24 (33.4%)	
	Mean± SD	Mean± SD	
Vitamin B12 (pg/ml)	345.33±103.22	308.42±94.63	0.135
HoloTC (pmol/l)	45.51±21.70	40.14±18.16	0.272

## Discussion

The correlation between hypothyroidism and vitamin B12 status in humans remains a topic of debate, and the evidence remains inconclusive despite extensive research, as certain studies suggested the presence of such a correlation [19] and other studies failed to establish such a correlation [20, 21, 22]. This correlation becomes more obscure when SCH is considered, owing to the scarcity of published studies on the matter [23].

The predominance of young females among the SCH patients in the present study aligns with the widely recognized higher prevalence of SCH among women. In a recent meta-analysis, it was observed that Hashimoto's thyroiditis exhibits a prevalence approximately four-fold higher among young females relative to their male counterparts [7, 24]. The emergence of sex differences in thyroid autoimmunity during puberty coincides with the simultaneous rise of leptin and estrogen levels. These hormones are known for their influence on the immune system and may play a significant role in the observed variations in disease incidence between genders [25]. Moreover, the mean BMI of subclinical hypothyroid patients was higher than that of apparently healthy controls. Hypothyroidism and obesity are intricately linked, as hypothyroidism is characterized by reduced resting energy expenditure [26]. Moreover, there is a higher prevalence of SCH observed among individuals who are obese [26-28]. The prevalence of positive anti-TPO antibodies in SCH correlates with autoimmune thyroiditis as the primary etiology of hypothyroidism [29]. Among patients with SCH, there is a notable propensity for progression to clinically overt hypothyroidism, occurring at a rate of 2.6% annually in those with negative anti-TPO antibodies and 4.3% in those with positive anti-TPO antibodies [8, 30].

The current study revealed a significant disparity in mean HoloTC levels between patients with SCH and apparently healthy controls, as there was a significantly lower mean HoloTC level among patients with SCH, while less pronounced variation was observed in mean vitamin B12 levels between these two groups, as there was an insignificantly lower mean vitamin B12 level among SCH patients. Although several studies have extensively explored the link between vitamin B12 deficiency and thyroid dysfunction, particularly overt hypothyroidism, investigations into this association with SCH remain limited [12, 31, 32]. Globally, different studies have reported a notable decrease in mean serum vitamin B12 levels among SCH patients compared to healthy subjects [33]. It is worth mentioning that neither of these studies incorporated or used serum HoloTC levels in assessing vitamin B12 status in patients with SCH [12]. The association between SCH and vitamin B12 deficiency can mostly be explained by the presence of the autoimmune process [34, 35]. Vitamin B12 deficiency highly arises from impaired absorption due to atrophic gastritis and pernicious anemia, which are frequently associated with autoimmune thyroid disease as a cluster of autoimmune disorders [36, 37].

The current study observed a crucial role of anti-TPO antibodies in vitamin B12 status in SCH, as the mean serum vitamin B12 level and mean serum HoloTC level were lower in SCH patients with positive anti-TPO antibodies compared to those with negative anti-TPO antibodies [38]. Therapeutic strategies for SCH patients commonly depend upon cutoff levels of TSH and the negativity or positivity of anti-TPO antibodies. A TSH level equal to or more than 7 µIU/L (33.4% of the patients with SCH) and positive anti-TPO antibodies (36.1% of the patients with SCH) among SCH patients is regarded as a potential indication for initiating T4 therapy, aiming to mitigate the risk of cardiovascular complications and progression to overt hypothyroidism [8]. The present study revealed a lower mean vitamin B12 and mean HoloTC level in SCH patients with TSH levels equal to or more than 7 µIU/L compared to those with TSH levels less than 7 µIU/L. Vitamin B12 deficiency in SCH patients exacerbates the risk of cardiovascular disorders, as vitamin B12 deficiency is a well-recognized cause of hyperhomocysteinemia that is considered a crucial risk factor for the development of cardiovascular diseases and their complications [39].

The strengths of the study include utilizing HoloTC along with vitamin B12 as markers for B12 deficiency; this study is the first to include both HoloTC and vitamin B12 in SCH, as well as, examining the correlation between HoloTC and vitamin B12 levels with the severity and autoimmunity of SCH.

The limitation of the study was that we chose SCH patients, which are a group of hypothyroid patients who are mostly asymptomatic, and their diagnosis depends on laboratory investigations performed more than once.

## Conclusions

In conclusion, this study sheds light on the association between serum vitamin B12 and HoloTC levels in SCH patients, particularly in relation to TSH levels and the presence of a higher level of anti-TPO antibodies. While significant differences were observed in serum TSH, anti-TPO antibodies, and HoloTC levels between SCH patients and healthy controls, the variance in vitamin B12 levels was not statistically significant. These findings underscore the potential impact of autoimmune processes and TSH levels on vitamin B12 status in SCH, warranting further investigation into the clinical implications of these relationships.

## Figures and Tables

**Figure 1:**
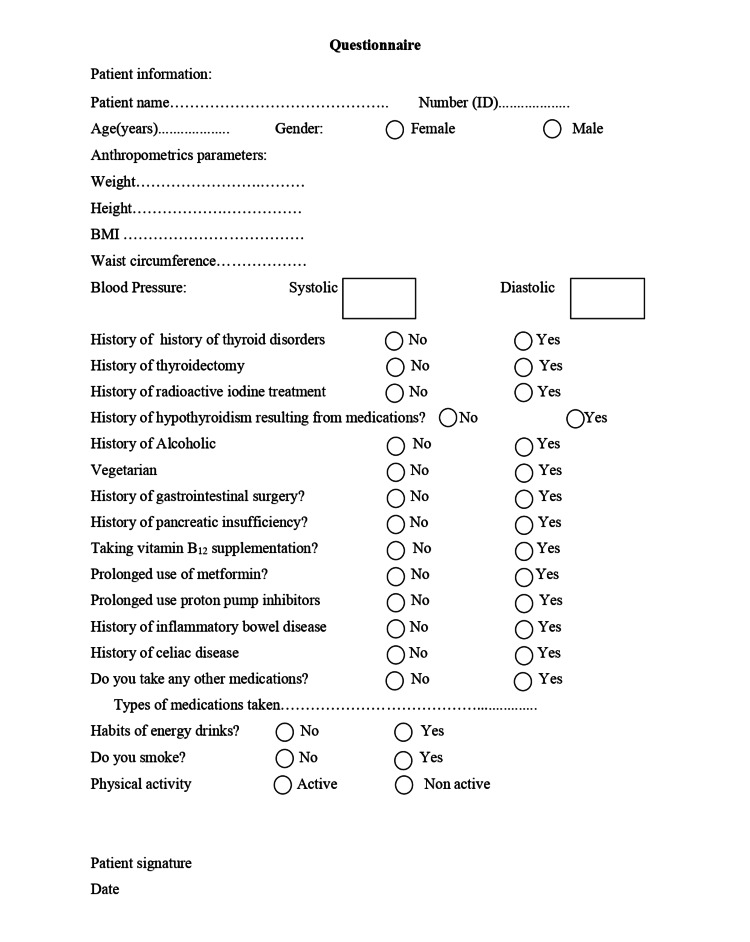
The questionnaire created by the authors that was distributed to all the study participants.

## Appendices

Appendix A 

The Questionnaire
